# A novel Gateway^®^-compatible binary vector allows direct selection of recombinant clones in *Agrobacterium tumefaciens*

**DOI:** 10.1186/1746-4811-7-42

**Published:** 2011-12-07

**Authors:** Sy Mamadou Traore, Bingyu Zhao

**Affiliations:** 1 Department of Horticulture, Virginia Polytechnic Institute and State University, Blacksburg, VA, 24061, USA

## Abstract

**Background:**

Cloning genes into plasmid vectors is one of the key steps for studying gene function. Recently, Invitrogen™ developed a convenient Gateway^® ^cloning system based on the site-specific DNA recombination properties of bacteriophage lambda and the cytotoxic protein ccdB, which is lethal to most *E. coli *strains. The ccdB protein, however, is not toxic to *Agrobacterium tumefaciens*, an important player often used for studying gene function *in planta*. This limits the direct application of the Gateway^® ^cloning system in plant transformation-mediated research.

**Results:**

In this study, we constructed a novel Gateway^®^-compatible destination vector, pEG101-SacB/R, by replacing the *ccdB *gene with a *SacB-SacR *gene cassette as the negative selectable marker.

**Conclusion:**

Our results demonstrate that the new pEG101-SacB/R destination vector can be used for Gateway^® ^cloning in *Agrobacterium tumefaciens*. pEG101-SacB/R will be a valuable tool for high-throughput functional analysis of genes *in planta*.

## Background

The wide-spread availability of genomic sequences from many organisms has raised interest in characterizing the biological functions of newly discovered genes through various high-throughput methodologies [1]. In order to study gene function, a gene-of-interest needs to be cloned into different plasmid vectors. In plant biology research, the gene-of-interest is frequently cloned into binary vectors that can be used for *Agrobacterium-*mediated transformation [2].

Gateway^® ^technology was developed as a convenient and fast gene cloning system (Invitrogen™, Carlsbad, CA). This method involves three key steps: (1) amplifying the targeted gene by PCR; (2) directly cloning the PCR product into a TopoEntr/D™ vector without digestion/ligation; and (3) subcloning the targeted gene from the entry vector into any destination vector using the Gateway^® ^LR cloning technique. The Gateway^® ^LR cloning reaction is mediated by the site-specific homologous DNA recombination properties of bacteriophage lambda [3]. This method is more convenient than other methods because it does not involve either DNA digestion nor ligation, two processes that can hinder the cloning process [4]. The Gateway^®^-compatible destination vector harbors a negative selectable marker, the *ccdB *gene, which produces a toxic protein that is lethal to most *E. coli *strains, including *DH5α *[5,6]. During the Gateway^® ^LR reaction, the *ccdB *gene in the destination vector is replaced by the targeted gene from an entry vector through site-specific homologous DNA recombination. The LR reaction mixture containing both recombinant and non-recombinant plasmids are subsequently transferred into an *E. coli *strain, such as *DH5α*, that is sensitive to the toxic effect of ccdB. Only recombinant destination plasmids that have lost the *ccdB *gene are able to survive in the transformed *E. coli *cells.

Recently, a collection of Gateway^®^-compatible binary destination vectors, that can be used for *Agrobacterium*-mediated transformation, have been constructed and are available to the plant research community [7-10]. Targeted genes can be easily cloned into these binary vectors through Gateway^® ^LR cloning. However, the Gateway^® ^LR cloning mixture has to first be transformed and screened in *E. coli *strains before the recombinant plasmid can be mobilized to *Agrobacterium tumefaciens *(*A*. *tumefaciens*) by either conjugation or electroporation. This process could be simplified if the recombinant binary plasmids from the Gateway^® ^LR reaction could be directly transformed and selected in *A*. *tumefaciens*. Unfortunately, *A*. *tumefaciens *is insensitive to the toxic effect of ccdB [6]. Thus, the binary vectors that contain the *ccdB *gene cannot be negatively selected against in *A*. *tumefaciens*. To overcome this problem, a new negative selectable marker that is functional in *A. tumefaciens *must be tested.

The *SacB-SacR *genes were originally isolated from *Bacillus subtilis *and encode levansucrase, an enzyme involved in both the hydrolysis of sucrose and the biosynthesis of levan [11,12]. Levan cannot be metabolized by most gram-negative bacteria, including *E. coli *and *A. tumefaciens*, and is therefore toxic to this group of organisms [13,14]. The *SacB-SacR *gene cassette, driven by its native promoter, has been used as a negative selectable marker for many gram negative bacteria and works by preventing the transformed bacterial strains from growing on culture medium supplemented with 5% sucrose [15]. Therefore, it will be interesting to test if the *ccdB *gene in any Gateway^®^-compatible binary vector can be replaced with the *SacB-SacR *gene cassette as the negative selectable marker. This would allow for direct transformation and screening of the Gateway^® ^LR cloning product in *A. tumefaciens*.

In this study, one popular Gateway^®^-compatible destination binary vector, pEarleyGate101 [9], was modified by replacing the *ccdB *gene with a *SacB-SacR *gene cassette. The novel destination binary vector, pEG101-SacB/R, would allow for the direct selection of Gateway^® ^recombinant clones in *A. tumefaciens*. To test the efficiency and functionality of pEG101-SacB/R, a *luciferase *gene (*Luc*) and a bacterial effector gene *Aae2166 *were used as reporters and cloned into the pEG101-SacB/R destination vector. The *Luc *gene encodes the fire fly luciferase protein, an enzyme that catalyzes luciferin and produces a luminescence signal when expressed in plant cells. This makes the *Luc *gene a convenient reporter gene for this study [16]. The bacterial type III putative effector gene *Aae2166*, isolated from bacterial pathogen *Acidovorax avenae *pv. *citrulli*, encodes a homolog of *AvrBsT *[17]. Transient expression of *Aae2166 *in the leaves of *Nicotiana benthamiana *(*N*. *benthamiana*) could trigger a cell death phenotype (Traore S and Zhao B, unpublished results). Therefore, *Aae2166 *is also a convenient marker for testing the functionality of the newly developed pEG101-SacB/R destination vector. The results of this study demonstrated that both genes were successfully cloned, selected for in *A. tumefaciens*, and expressed in *N. benthamiana *leaves. Therefore, the new destination vector is a convenient tool for cloning and expressing genes in *A. tumefaciens*. The new vector also has potential to be used in high-throughput cloning applications *in planta*.

## Methods

### Bacteria strains

*Escherichia coli *(*E. coli*) *DH5α *[*F^- ^endA glnV44 thi-1 recA1 relA1 gyrA96 deoR nupG Φ80dlacZΔM15Δ(lacZYA-argF)U169, hsdR17(r_K_^-^m_K_^+^), λ-*] and *A. tumefaciens *(GV2260) [C58 background, rifampicin resistant with the Ti plasmid (pTiB6s3)]

### Preparation of electroporation competent cells

*1*. *E. coli DH5α *and *A. tumefaciens GV2260 *strains were streaked on Luria Broth (LB) agar media supplemented with appropriate antibiotics and incubated at 37°C and 28°C, respectively.

*2*. Single colonies of *DH5α *and *A. tumefaciens *(*GV2260*) were inoculated in 50 ml of LB liquid media, incubated at 37° and 28°C, respectively, and shaken at 200 rpm for 24 hours.

*3*. A new 500 ml LB liquid culture was re-inoculated from the initial saturated bacterial culture to produce a final concentration of OD_600 nm _= 0.1. The liquid cultures were incubated at 18°C and 28°C for *DH5α *and *A. tumefaciens (GV2260)*, respectively, until the OD at 600 nm reached 0.6.

*4*. The bacterial liquid culture was subsequently chilled on ice for 20 minutes.

*5*. Bacterial cells were harvested by centrifugation at 6000 × g for 10 minutes at 4°C.

*6*. The bacterial cells were washed twice with 250 ml of ice-cold water, and then finally washed with 25 ml of 10% ice-cold glycerol.

*7*. The bacterial cells were pelleted by centrifugation and re-suspended in 5 ml of 10% ice-cold glycerol.

*8*. A 100 μl aliquot of bacterial cells was transferred to 1.5 ml microtubes and immediately frozen in liquid nitrogen.

*9*. The cells were stored at -80°C for at least six months without loss of competency.

### Plant materials

*N. benthamiana *(PI 555478) plants were propagated in a growth chamber programmed for 16 hours light (140 μmol/m^2^/s cool white fluorescent irradiance) at 28°C and 8 hours dark at 24°C. *Agrobacterium*-mediated transient assays were conducted on three to four week old plants.

### Construction of the destination binary pEG101-SacB/R vector

The *SacB-SacR *genes were amplified by PCR from plasmid pMsacB [18] using primers SacB/R Forward: 5'-AATCAGGAAGGGATG**GCTGAGG**GATATCGGATCGATCCTTTTTAACCCATCAC-3'and SacB/R Reverse: 5'-AGACCGGCACACTGGCCATATCGGTGGGATATCTTATTTGTTAACTGTTAATTGTCCT-3'. The *Bbv*CI and *Bst*XI restriction sites appear in the primers as bolded and underlined text, respectively. The nucleotide sequences of the *SacB-SacR *genes, including their native promoter, can be found in GenBank [GenBank:AB183144].

The iProof ™ high fidelity Taq DNA polymerase (Bio-Rad, Hercules, CA) PCR program consisted of 1 cycle at 98°C (2 min), followed by 30 cycles at 98°C (30 s), 55°C (45 s), and 72°C (1 min), and finished with a 1 cycle extension at 72°C (7 min). The PCR products were digested with *Bbv*CI and *Bst*XI (New England BioLabs Inc, Ipswich, MA), and gel purified using an AccuPrep™ Gel Purification Kit (Bioneer, Alameda, CA).

The destination binary plasmid pEarleyGate101 [9] was obtained from the Arabidopsis Biological Resource Center at Ohio State University (Columbus, OH) and digested with *Bbv*CI and *Bst*XI to remove the *ccdB *gene. The gel-purified PCR product of the *SacB-SacR *gene cassette was then cloned into the *Bbv*CI and *Bst*XI restriction sites of pEarleyGate101. The ligation products were transformed into *DH5α *using a gene pulse electroporation apparatus (2.5 Kv/uM/mSec) (Bio-Rad). Successfully transformed bacteria were selected for on LB medium supplemented with kanamycin (50 mg/ml) and chloramphenicol (25 mg/ml). The derived new destination vector was re-named pEG101-SacB/R.

### Plasmid DNA isolation

All plasmid DNA was isolated using an AccuPrep™ Plasmid Extraction Kit (Bioneer).

### Negative selection for the *SacB-SacR *gene

Bacterial cells that were successfully transformed with the pEG101-SacB/R plasmid were negatively selected for on LB medium supplemented with 5% sucrose as described previously [13,14].

### Cloning of the *Luc *gene and *Aae*2166 into the TopoEntry/D vector

The open reading frame (ORF) of the *Luc *gene was cloned from plasmid pDesA-luc [19] with primers: Sal_LucFor:5'-caccgtcgacGGCGGTGGCTCATCTGGCGGAGGT**atg**gaagacgccaaaaac-3' and Pst_LucRev: 5'-ctgcag**TTA**CAATTTGGACTTTCCGCCCTTCTTGG-3'. The start and stop codons of the *Luc *gene are bolded and underlined in the forward and reverse primers, respectively. The ORF of the *Aae2166 *gene was amplified from genomic DNA of *Acidovorax avenae *subs *citrulli *strain AAC-001 using primers: Aae2166 For: 5'-caccAGATCT**ATG**AAGAATTTCATGCGATCGAT -3' and Rev: 5'- GTCGACTTCGATAGCTTTTCTGATTTTTCTCA-3'. The start codon of Aae2166 is bolded and underlined. The PCR products of the *Luc *and *Aae2166 *genes were gel purified and cloned into the TopoEntr/D™vector (Invitrogen) following the instructions of the user's manual. Plasmid DNA was sequenced at the Core facility of the Virginia Bioinformatics Institute (Blacksburg, VA).

### LR reaction and transformation of *Agrobacterium tumefaciens *by electroporation

The Gateway^® ^LR clonase enzyme mix kit (Invitrogen) was used for the LR recombination reaction. In brief, the LR reaction mixture contained: 1-5 μl (100 ng) of entry clone plasmid DNA, 1 μl (100 ng) of destination vector pEG101-SacB/R plasmid DNA, 2 μl of 5 × LR clonase reaction buffer, and 2 μl of LR clonase enzyme. The reaction mixture was brought to a final volume of 10 μl with TE buffer (pH 8.0) and incubated at room temperature for 1 hour. Following the incubation, a 3-5 μl aliquot was mixed with 100 μl of competent *A. tumefaciens *(GV2260) cells. A gene pulse apparatus (Bio-Rad), programmed for 2.5 Kv/uM/mSec, was used to transform the cells by electroporation. Following electroporation, the mixture was resuspended in 1 ml of liquid LB and incubated shaking at 28°C/200 rpm for 3 hours. Successfully transformed cells were then selected for on LB agar supplemented with kanamycin (50 mg/ml), rifampicin (100 mg/ml), and 5% sucrose. The LB plates were incubated at 28°C for 2-3 days. After incubation, ten colonies were randomly selected for further analysis. Recombinant plasmids were confirmed by PCR with primers: 35S forward 5'-AAGAAGACGTTCCAACCACGTC-3' and NLuc reverse: 5'-AACTGCAGTCATCCATCCTTGTCAATCAAG-3' to detect the *Luc *gene (1.4 kb) [19]. Recombinant plasmids were also confirmed by PCR using primers 35S forward plus Aae2166 reverse: 5'- GTCGACTTCGATAGCTTTTCTGATTTTTCTCA-3' to detect the *Aae2166 *gene (1.1 kb). The PCR fragments were separated on a 0.8% agarose gel, stained with 0.01% ethidium bromide solution, and visualized using the Gel-Document Image System™ under UV light (Bio-Rad).

### *Agrobacterium*-mediated transient assays in *N. benthamiana *plants

*Agrobacterium*-mediated transient assays in *N. benthamiana *plants were performed as described previously [20]. In brief, the *Agrobacterium *strain was streaked on LB medium supplemented with appropriate antibiotics and incubated at 28°C for 2 days. Bacterial cells were harvested and re-suspended in induction buffer composed of 10 mM MgCl_2_, 10 mM MES (pH 5.6), and 100 μM acetosyringone and incubated for 3 hours at room temperature. The bacterial inoculums were adjusted to OD_600 nm _= 0.6 and infiltrated into the stomata of fully expanded *N. benthamiana *leaves using a 1 ml blunt-end syringe without a needle. The inoculated plants were incubated at room temperature under continuous light for 20-48 hours before the detection of expressed proteins. Transient expression of the *Luc *gene was detected by applying 1 μM luciferin to the inoculation site [21]. The chemical fluorescent signals were captured with a CCD camera and visualized using the Gel-Document Image System (Bio-Rad). The fluorescent signal of the Aae2166-GFP fusion protein was monitored 20 hours after inoculation by fluorescent microscopy (Zeiss Axio Observer.A1, Carl Zeiss MicroImaging, Inc., Thornwood, NY).

## Results and discussion

### Constructing a novel Gateway^®^-compatible binary vector pEG101-SacB/R

**Figure 1:**
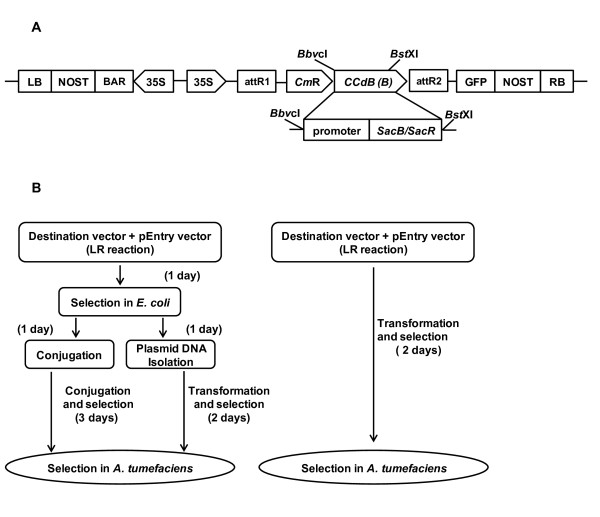
**Schematic cloning of a gene into a binary expression vector and selection in *Agrobacterium tumefaciens***. (A) Key features of pEG101-SacB/R. LB: T-DNA left border; RB: T-DNA right border; *Cm*^R^: chloramphenicol resistance gene; attL1 and attL2: recognition sites of the LR clonase; *ccdB*: the Invitrogen *ccdB *gene cassette frame B; *SacB/SacR*: Levansucrase genes along with their native promoter; *Bbv*CI and *Bst*XI: restriction enzyme sites for replacing the *ccdB *gene fragment with the *SacB/SacR *gene cassette. (B). Compared to the regular LR cloning procedure, the new protocol is more convenient and time-saving.

**Figure 2:**
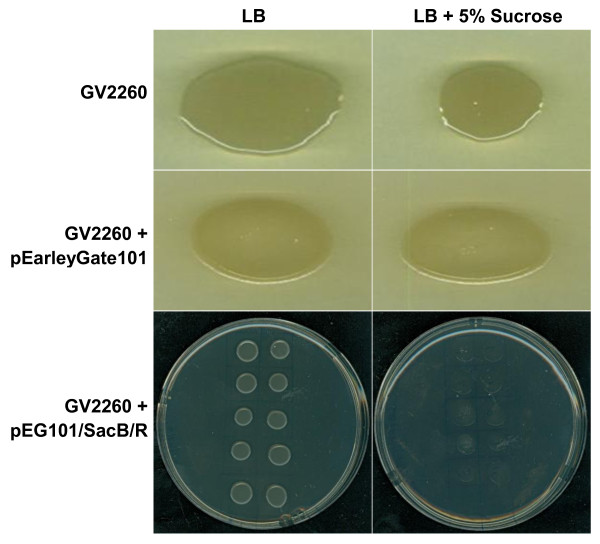
**The growth of *Agrobacterium tumefaciens *strain on LB agar plates with different selections**. *A. tumefaciens *strain GV2260 grew equally well on LB medium with (A) or without 5% sucrose (B). The *ccdB *gene cassette in the pEarleyGate 101 vector could not inhibit the growth of *A. tumefaciens *on LB medium with (C) or without 5% sucrose (D). *A. tumefaciens *strain GV2260 carrying the newly generated pEG101-SacB/R destination vector grew well on LB medium without sucrose (E), but growth was inhibited on LB medium supplemented with 5% sucrose (F).

In order to construct a new Gateway^®^-compatible destination binary vector that can be used for selection of recombinant plasmids in *A. tumefaciens*, the *SacB-SacR *gene cassette was amplified and cloned into the *Bbv*CI and *Bst*XI restriction sites of pEarleyGate 101 [9]. The *SacB-SacR *genes replaced *ccdB *to generate a novel destination vector, pEG101-SacB/R. The key components of pEG101-SacB/R are illustrated in Figure 1A. To test the functionality of the *SacB-SacR *gene cassette as an effective negative selectable marker, *A. tumefaciens *strain GV2260, *A. tumefaciens *strain GV2260 carrying pEarleyGate101, and *A. tumefaciens *strain GV2260 carrying pEG101-SacB/R were grown on LB agar medium supplemented with or without 5% sucrose (Figure 2). As shown in Figure 2A and 2B, *A. tumefaciens *grew equally well on LB medium with or without 5% sucrose, which suggested that sucrose in LB agar medium has no inhibitory effect to *A. tumefaciens*. The *A. tumefaciens *strain GV2260 carrying pEarleyGate101 also grew well on LB agar medium with or without 5% sucrose (Figure 2C and 2D). This result demonstrates that the *ccdb *gene cassette in pEarleyGate101 cannot be used as a negative selectable marker in *A. tumefaciens*. To test the functionality of the *SacB-SacR *gene cassette as an effective negative selectable marker in *A. tumefaciens*, the pEG101-SacB/R plasmid DNA was transformed into *A. tumefaciens *strain GV2260. After transformation, ten randomly selected clones were raised on LB medium supplemented with or without 5% sucrose. *A. tumefaciens *carrying pEG101-SacB/R grew well on LB agar medium without sucrose (Figure 2E); however, their growth was completely inhibited when the LB agar medium was supplemented with 5% sucrose (Figure 2F). These results show that the new destination vector, pEG101-SacB/R, can efficiently select against *A. tumefaciens *when grown on LB agar medium supplemented with 5% sucrose.

### Testing the stability of binary vector pEG101-SacB/R

**Figure 3:**
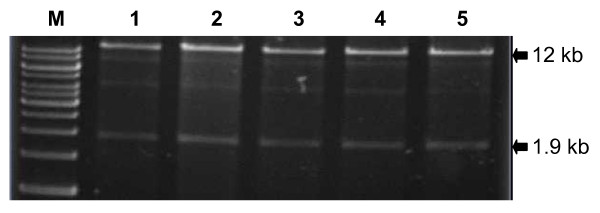
**The destination vector pEG101-SacB/R can be stably propagated in the *E. coli *strain *DH5α***. Restriction digestion (*Bst*XI and *Bbv*CI) of plasmid DNAs, isolated from five repeated overnight cultures of *E. coli *carrying the pEG101-SacB/R destination vector, showed identical restriction patterns. M: 1 kb marker. Lanes 1 through 5 were digested plasmid DNAs of five repeated subcultures.

To test the stability of destination vector pEG101-SacB/R in *E. coli*, the restriction patterns of pEG101-SacB/R were compared before and after repeated sub-culturing. A single *E. coli *clone carrying pEG101-SacB/R was grown overnight in LB liquid medium supplemented with both kanamycin and chloramphenicol. The overnight bacterial culture was diluted (5000 ×) with LB medium and sub-cultured for an additional 24 hours. This process was repeated five times as described previously [22]. The plasmid DNA of pEG101-SacB/R was isolated after each generation and digested with *Bst*XI and *Bbv*CI to remove a 1.9 kb fragment corresponding to the inserted *SacB-SacR *gene cassette. As shown in Figure 3, the restriction digestion patterns of pEG101-SacB/R from each generation were identical. All plasmid DNAs were further transformed into both *E. coli *and *A. tumefaciens *and the derived clones were re-plated on LB agar medium supplemented with or without 5% sucrose. All transformed bacteria colonies failed to grow on LB agar medium supplemented with 5% sucrose, confirming the functionality of the *SacB-SacR *genes. These results demonstrate that pEG101-SacB/R can be stably propagated in *E. coli *grown on LB agar medium without sucrose.

### Generating recombinant clones in *Agrobacterium tumefaciens *through Gateway LR cloning

**Figure 4:**
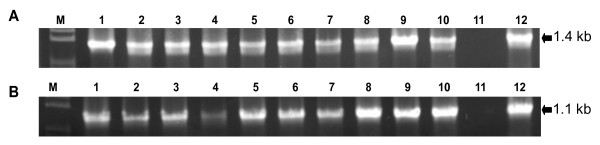
**PCR analysis of recombinant clones of *Agrobacterium tumefaciens***. (A). Clones of *A. tumefaciens *transformed with pEG101-*Luc*. M: 1 kb marker; lanes 1 through 10 were from bacteria colonies carrying the recombinant plasmid pEG101-*Luc*, where a 1.4 kb band was amplified corresponding with the expected 35S-*Luc *gene; lane 11 was a negative PCR control using *A. tumefaciens *only as a template; lane 12 was a positive PCR control using plasmid DNA of pEG101-*Luc *isolated from *E. coli *as a template. (B). Clones of *A. tumefaciens *transformed with pEG101-*Aae2166*. M: 1 kb marker; lanes 1 to 10 were clones carrying the recombinant plasmid pEG101-*Aae2166*. Amplified fragments corresponded to 35S-*Aae2166 *(1.1 kb). Lane 11 was a negative PCR control using *A. tumefaciens *only as a template. Lane 12 was a positive control using plasmid DNA of pEG101-*Aae2166 *isolated from *E. coli *as a template.

The functionality of pEG101-SacB/R for Gateway^® ^LR cloning was tested in *A. tumefaciens *using two entry clones carrying the *Luc *and *Aae2166 *genes, respectively. *A. tumefaciens *strain GV2260 was directly transformed with the LR reaction mixture for each gene by electroporation. Putative recombinant clones were selected on LB agar medium supplemented with kanamycin (50 mg/ml) and 5% sucrose. Ten randomly selected *A*. *tumefaciens *clones were analyzed by PCR using either *Luc *or *Aae2166 *gene specific primers, or a combination of gene specific primers with a primer designed using the 35S promoter in the vector pEG101-SacB/R as a template (Figure 1A). As shown in Figure 4, all colonies tested were positive, suggesting a high cloning efficiency of targeted genes into pEG101-SacB/R in *A*. *tumefaciens*. We also used the vector to clone twenty two bacterial type III effector genes of *Acidovorax avenae pv. citrulli*, which gave a similar cloning efficiency (data not shown).

As shown in Figure 1A, the cloned *Luc *and *Aae2166 *genes are driven by the 35S promoter. The ORF of *Aae2166 *was cloned, without a stop codon, into the pEntry/D vector. After LR cloning, the lack of a stop codon allowed the *Aae2166 *ORF to be fused in-frame with the N-terminus of the GFP gene in the recombinant pEG101-*Aae2166 *plasmid. Conversely, a stop codon was added to the C-terminus of the *Luc *gene; therefore, GFP fusion would not have occurred.

### Validating the function of recombinant binary plasmids by *Agrobacterium*-mediated transient assays

**Figure 5:**
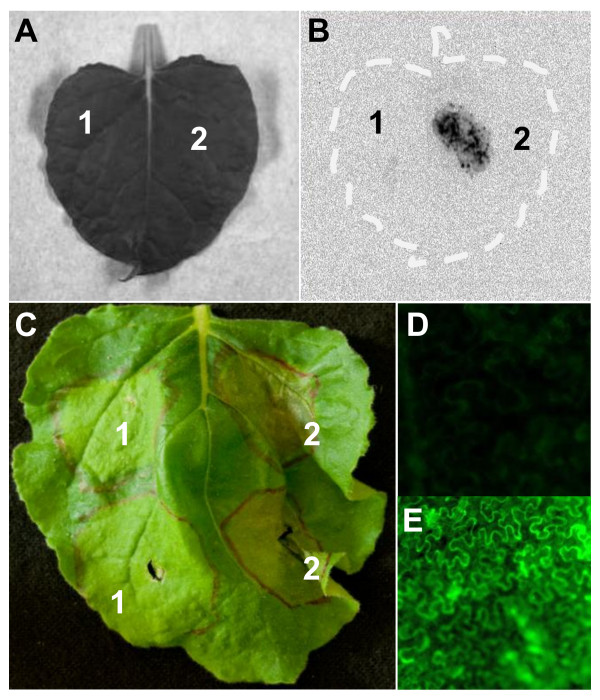
**Functionality test of two genes, cloned into the pEG101-SacB/R destination vector, by means of *Agrobacterium*-mediated transient assays in the leaves of *Nicotiana benthamiana***. *A. tumefaciens *GV2260 strains, carrying either pEarleyGate 101 empty vector (A-1) or pEG101-*Luc *(A-2), were inoculated in *N. benthamiana *leaves. The *A. tumefaciens *GV2260 strain carrying pEG101-*Luc *(B-2) produced a strong luminescent signal under dark conditions after luciferin treatment, while the strain carrying the unmodified vector pEarleyGate 101 (B-1) did not show any signal. The luminescent signal was detected using the Gel Document Image System equipment with a CCD camera. *A. tumefaciens *strain GV2260 carrying pEG101-*Aae2166 *(C-2), but not the empty vector pEarleyGate 101 (C-1), was able to trigger programmed cell death in the leaf of *N. benthamiana*. The fluorescent signal of the Aae2166-GFP fusion protein was detected by fluorescent microscopy (200 × magnifications) (E). As the negative control, GFP signal was not detected in the leaf of *N. benthamiana *that was inoculated with *A. tumefaciens *carrying pEarleyGate 101 (D).

To examine the expression of the *Luc *and *Aae2166 *genes cloned in pEG101-SacB/R, *A. tumefaciens *colonies carrying the recombinant plasmids pEG101-*Luc *or pEG101-*Aae2166 *were inoculated into the leaves of *N. benthamiana*. As shown in Figure 5B, *A. tumefaciens *colonies carrying pEG101-*Luc *(Figure 5 B-2) but not pEarleyGate 101 (Figure 5 B-1) triggered strong luminescence after treatment with luciferin, thereby confirming the expression of *luciferase*. As a negative control, the same *A. tumefaciens *strain, carrying pEG101-*Luc*, was spotted onto either the leaf surface of *N. benthamiana *or onto a LB agar plate. After treatment with luciferin, the samples were examined under a CCD camera and no luminescence signal was detected (data not shown). This result confirmed that the luminescence signal in Figure 5B-2 was from the transiently transformed *N. benthamiana *plant cells. When *N. benthamiana *plants were inoculated with *A. tumefaciens *colonies carrying pEG101-*Aae2166*, cell death was observed at 48 hours after inoculation (Figure 5C-2). As a negative control, an *A. tumefaciens *strain carrying an un-modified pEarleyGate101 was inoculated into the leaves of *N*. *benthamiana*, which did not trigger cell death at 48 hours after inoculation (Figure 5C-1). Since we modified Aae2166 to contain a C-terminal GFP tag (Figure 1A), pEG101-*Aae2166 *expressed an Aae2166-GFP fusion protein. As shown in Figure 5E, the fluorescent signal of the Aae2166-GFP fusion protein was visible by fluorescent microscopy. The picture in Figure 5E was taken at 20 hours after inoculation, at which point there was no cell death observed at this time. This eliminated the possibility of autofluorescent signals from dead cells. Aae2166-GFP proteins were predominantly localized in the cytosol of transformed cells, although this observation requires further confirmation by an independent method. As a negative control, *A. tumefaciens *carrying an un-modified pEarleyGate101 did not incite a fluorescent signal in *N*. *benthamiana *(Figure 5D). These results demonstrated that two different genes could be efficiently cloned into pEG101-SacB/R using the Gateway LR reaction with direct selection in *A. tumefaciens*. The two genes were functionally expressed when assayed by *Agrobacterium*-mediated transient assays.

## Conclusions

In this report, a novel Gateway^®^-compatible binary vector pEG101-SacB/R is described that allows direct transformation and selection of recombinant LR-generated clones in *A. tumefaciens*. The *Luc *gene, along with a putative bacterial effector gene, *Aae2166*, were used to demonstrate the efficiency and functionality of this newly developed Gateway^®^-compatible binary vector. The ability to directly clone and select recombinant genes in *A. tumefaciens *would save time, labor, and would also minimize potential contamination problems associated with conjugation or transformation manipulation. The new vector pEG101-SacB/R described here has great potential for simplifying and improving the efficiency of cloning genes in *A. tumefaciens *for plant transformation research. The pEG101-SacB/R vector can also be adapted for high- throughput applications. For example, a gene-of-interest mutant library could be generated by Error-Prone PCR from a TopoEntr/D™ (Invitrogen) plasmid template. The loss-of-function mutants could be identified through *Agrobacterium*-mediated transient assays in *N. benthamiana *leaves. Therefore, the new destination binary vector pEG101-SacB/R may be a valuable tool for high-throughput functional analysis of genes *in planta*.

## Competing interests

No competing interests were claimed by the authors. The vector pEG101-SacB/R is available upon request from the authors.

## Authors' contributions

ST designed and performed the experiments. BZ is the the principal investigator who conceived the project and the cloning strategy. ST and BZ analyzed the data and wrote the manuscript. All authors read and approved the final manuscript.
